# 5-Azacytidine treatment sensitizes tumor cells to T-cell mediated cytotoxicity and modulates NK cells in patients with myeloid malignancies

**DOI:** 10.1038/bcj.2014.14

**Published:** 2014-03-28

**Authors:** A O Gang, T M Frøsig, M K Brimnes, R Lyngaa, M B Treppendahl, K Grønbæk, I H Dufva, P thor Straten, S R Hadrup

**Affiliations:** 1 Department of Hematology, Center for Cancer Immune Therapy, University Hospital Herlev, Herlev, Denmark; 2 Department of Drug Design and Pharmacology, Faculty of Health and Medical Sciences, University of Copenhagen, Copenhagen, Denmark; 3 Department of Hematology, Rigshospitalet, Copenhagen, Denmark; 4 Department of Hematology, University Hospital Herlev, Herlev, Denmark

## Abstract

Treatment with the demethylating agent 5-Azacytidine leads to prolonged survival for patients with myelodysplastic syndrome, and the demethylation induces upregulation of cancer-testis antigens. Cancer-testis antigens are well-known targets for immune recognition in cancer, and the immune system may have a role in this treatment regimen. We show here that 5-Azacytidine treatment leads to increased T-cell recognition of tumor cells. T-cell responses against a large panel of cancer-testis antigens were detected before treatment, and these responses were further induced upon initiation of treatment. These characteristics point to an ideal combination of 5-Azacytidine and immune therapy to preferentially boost T-cell responses against cancer-testis antigens. To initiate such combination therapy, essential knowledge is required about the general immune modulatory effect of 5-Azacytidine. We therefore examined potential treatment effects on both immune stimulatory (CD8 and CD4 T cells and Natural Killer (NK) cells) and immune inhibitory cell subsets (myeloid-derived suppressor cells and regulatory T cells). We observed a minor decrease and modulation of NK cells, but for all other populations no effects could be detected. Together, these data support a strategy for combining 5-Azacytidine treatment with immune therapy for potential clinical benefit.

## Introduction

5-Azacytidine is a cytosine analog and a potent DNA methyltransferase inhibitor, previously shown to induce DNA demethylation. Treatment with 5-Azacytidine (Vidaza, Celgene Corporation, Boudry, Switzerland) is used for patients with higher-risk myelodysplastic syndrome (MDS),^1, 2^ and for a subgroup of acute myeloid leukemia (AML)^3^ and chronic myelomonocytic leukemia (CMML)^4^ patients. 5-Azacytidine induces a late clinical response in some patients,^2, 5, 6^ and this has led to speculations that immune-mediated mechanisms could be involved, as immune modulatory interventions often have slower onset of efficacy than direct cytotoxic drugs.^7^ It has been shown that 5-Azacytidine upregulates cancer-testis antigen (CTA) expression in tumor cells as a result of demethylation.^8, 9, 10^ This upregulation may increase immune recognition of tumor cells as CTAs are well-known targets for immune recognition in cancer.^11, 12, 13^ They are of special interest because of their very restricted expression pattern in healthy tissues, involving primarily immune-privileged sites, such as testis, placenta and during fetal development.^14, 15, 16, 17^

In the present study, we investigated whether 5-Azacytidine treatment increased the direct tumor cell recognition by host T cells to provide a direct link to tumor cell killing not biased by antigen selection or HLA expression. CD8 T cells and autologous myeloid blasts were isolated from peripheral blood at different time points, separated and rested before re-exposure of tumor cells to T cells to assess their recognition through upregulation of CD107a expression. Furthermore, we analyzed whether single-therapy treatment with 5-Azacytidine induced T-cell responses against CTA-derived epitopes, as previously observed in combination with histone deacetylase inhibition treatment.^10^ We analyzed for specific T-cell responses against a panel of 43 CTA-derived epitopes restricted to HLA-A1, -A2, -A3 and -B7^18^ to extent the diversity of previously observed responses. These were detected through combinatorial encoded major histocompatibility complex (MHC) class I multimers in a flow cytometry-based approach.^19^ Induced immune recognition of tumor cells and increased CTA-specific T-cell responses during therapy would speak for the combination of 5-Azacytidine and CTA-specific immune therapeutic strategies. A number of other chemotherapeutic regiments has been shown to modulate the immune system in a favorable manner to increase antitumor immunity.^20^

To potentially combine 5-Azacytidine with immune therapy, it is essential to understand any functional impact of 5-Azacytidine directly on immune stimulatory and inhibitory cell subsets. In particular, the Natural Killer (NK)-cell subset has previously been of interest in relation to the development and prognosis of AML and MDS. The absolute counts and activity of NK cells are reduced in leukemic patients, and low NK cell counts are associated with poor prognosis.^21, 22^ In addition to NK cells, CD4 and CD8 T cells are of major importance in the adaptive immune system. We investigated 5-Azacytidine's impact on functionality and frequency of CD4 and CD8 T cells and NK cells. The effect of 5-Azacytidine on NK-cell function has previously been the focus of several *in vitro* studies that showed impaired function of NK cells during treatment. This impairment was due to overexpression of inhibitory NK receptors, reduced cytokine mRNA synthesis and enhanced NK-cell apoptosis.^23, 24^ However, the *in vivo* impact of 5-Azacytidine on the NK-cell population has to our knowledge never been investigated.

Moreover, effects of 5-Azacytidine on the immune regulatory myeloid-derived suppressor cells (MDSCs) and regulatory T cells (Tregs) were investigated as these are key factors inhibiting antitumor immunity.^25, 26^ Accumulation of both cell populations correlates with poor prognosis in many cancers, including MDS.^26, 27^ Tregs are additionally of particular interest in relation to 5-Azacytidine treatment as mouse studies has shown induced expression of the transcription factor FOXP3 on naive T cells by the 5-Azacytidine deoxyribonucleoside analog decitabine. This induction transformed naive T cells both phenotypically and functionally into a regulatory subset, contributing to cytotoxic T-cell suppression.^28^

The immunological impact of 5-Azacytidine was evaluated on a diverse cohort of MDS, AML and CMML patients. Peripheral blood was collected and analyzed before and throughout therapy. Together these results signify the feasibility of combining 5-Azacytidine with immune therapeutic strategies.

## Materials and methods

### Patients

Seventeen patients, 10 diagnosed with high-risk MDS or MDS with high-risk features, 4 with AML, 1 with MDS/AML and 2 with CMML, were treated with 100 mg/m^2^ s.c. 5-azacytidine daily for 5 days every fourth week for at least three cycles at University Hospital Herlev, Denmark. Distance between cycles could be increased on the treating physicians' request due to treatment toxicity or slow bone marrow recovery. The protocol was approved by the institutional ethical committees, Copenhagen County and Danish Medicine's Agency. All patients gave written informed consent according to the Helsinki Declaration before study entry. Peripheral blood (50 ml) was collected twice during each cycle and processed for later analysis. Patient characteristics are given in Table 1, and further Material and Methods details according to the MIATA (Minimal Information About T-cell Assays) guideline are provided in Supplementary Table S1.

### PBMC collection and processing

Peripheral blood mononuclear cells (PBMCs) were isolated from peripheral blood by gradient centrifugation (Lymphoprep, 1.077 g/ml, Nycomed Pharma AS, Oslo, Norway) at room temperature within a few hours after the blood was obtained from the patient. PBMCs were cryopreserved in 90% heat-inactivated fetal calf serum (FCS, Gibco, Life Technologies, Naerum, Denmark) and 10% dimethyl sulfoxide (Sigma-Aldrich, Broendby, Denmark) with 5–30 × 10^6^ cells/ampoule and stored at −150 °C. All analyses were conducted on cryopreserved material. Manual cell counting was done using trypan blue (Sigma-Aldrich) staining.

### Intracellular cytokine staining

CD34 myeloid blasts and CD8 T cells were separated from PBMCs by magnetic bead-based separation (MACS, Miltenyi Biotec, Bergisch Gladbach, Germany). CD34 cells were separated using positive selection, whereas CD8 T cells were isolated from the CD34-negative fraction using negative selection, according to the manufacturer's instructions. The yield of these isolations varied greatly between patients. The two cell populations were rested separately overnight at 37 °C and 5% pCO_2_ in X-vivo 15 (Lonza, Vallensbaek, Denmark), 10% human serum (Sigma-Aldrich) and co-cultured at effector:target (E:T) ratio: 1:2, 1:5 or 1:10 for measuring cytotoxicity via extracellular expression of CD107a. This cytotoxicity assay was done using CD34 cells from first cycle (day 1, before treatment) or CD34 cells from a late cycle (4th–6th cycle), mixed separately with CD8 T cells from first cycle or CD8 T cells from a late cycle. This setup resulted in four different co-cultures per patient: CD34 first and CD8 first, CD34 first and CD8 late, CD34 late and CD8 first, and CD34 late and CD8 late. Whenever possible we used 1:10 E:T ratio, but for some patients low numbers of isolated cells required a lower ratio (AZA 4 and AZA 27: 1:2, AZA 28: 1:5, AZA 14: 1:10 in first cycle tumor co-cultures and 1:2 in late cycle tumor co-cultures), all paired analyses were done with the same E:T ratio. After co-culture, the CD107a expression was measured after 5 h of incubation at 37 °C and 5% pCO_2_, as previously described.^29^ Further information is provided in the Supplementary Material. Samples were compared over time, and no additional negative or positive controls were included because of limited material.

### Detection of specific T-cell populations

Antigen-specific T cells were stained directly *ex vivo* or after a peptide pre-stimulation step by combinatorial encoded MHC multimers and analyzed by flow cytometry.

*In vitro* peptide pre-stimulation: T cells were stimulated *in vitro* with peptides for 7 days at 37 °C and 5% pCO_2_ in X-vivo 15 (Lonza) with 5% human serum (Sigma-Aldrich), IL-2 (20 U/ml, Proleukin, Novartis Healthcare, Copenhagen, Denmark) and IL-7 (5 ng/ml, Peprotech Nordic, Stockholm, Sweden). Peptides were pulsed on the PBMCs in 10 μM for 4 h at room temperature and washed before culture. After 7 days, the frequency of MHC-multimer-specific T cells was determined by flow cytometry. Negative controls were included in the MHC-multimer staining, and details of the MHC-multimer-based flow cytometry analyses are provided in the Supplementary Material and was described in detail recently.^30, 31, 32^

### T-cell and NK-cell functionality assays

PBMCs where thawed in RPMI Medium 1640+GlutaMax (Sigma-Aldrich) with 10% FCS (both from Gibco, Life Technologies, Naerum, Denmark) and DNase (Invitrogen, Life Technologies, Naerum, Denmark), and rested overnight in 24-well plates at 37 °C and 5% pCO_2_ in X-vivo 15 (Lonza) with 10% human serum (Sigma-Aldrich). To activate the T cells, we used the superantigen *Staphylococcus aureus* enterotoxin B (Sigma-Aldrich). In the negative control nothing was added. For the NK cell-mediated killing, the MHC class I-deficient cell line K562 served as target cells, E:T ratio was 1:1. CD107a-PE (BD Pharmingen, Albertslund, Denmark) and BD GolgiPlug (BD Biosciences, Albertslund, Denmark) were added and cells incubated for 5 h according to the CD107a assay protocol.^29^ PBMC samples were surface stained with CD3-PerCP-Cy5.5, CD16-HV500 (both from BD Pharmingen), CD56-BV421 (BioLegend, Nordic Biosite, Copenhagen, Denmark) and LIVE/DEAD Fixable Near-IR Dead Cell Stain Kit for 633 or 635 nm excitation (Invitrogen, Life Technologies) for the NK cells and CD3-PerCP-Cy5,5, CD8-HV450, CD4-HV500 (all from BD Pharmingen) and LIVE/DEAD Fixable Near-IR Dead Cell Stain Kit for 633 or 635 nm excitation (Invitrogen, Life Technologies) for the T cells. Acquisition was conducted on a FACSCanto II (BD Biosciences) and data analyzed using FACSDiva software (BD Biosciences).

### NK-cell killing capacity assay

CD3^−^CD56^+^ NK cells were isolated from PBMCs from two healthy donors using MACS-untouched NK isolation kit followed by MACS CD56-positive selection kit (both Miltenyi Biotec) to purify the population (data not shown), and rested overnight at 37 °C and 5% pCO_2_ in X-vivo 15 (Lonza) with 10% human serum (Sigma-Aldrich), IL-2 (200 U/ml, Proleukin, Novartis, Copenhagen, Denmark) and IL-15 (40 U/ml, Peprotech Nordic, Stockholm, Sweden). The yield varied greatly between donors. On the following day, the NK cells were divided equally in wells in a 48-well plate (Corning Costar, BD Biosciences), and 5-Azacytidine (Sigma-Aldrich) was added in 0 nM, 0.88 nM, 2500 nM or 5000 nM. Total volume in each well was 200 μl. On days 2 and 3, the cells were either supplemented with new 5-Azacytidine or not, all cells were re-suspended every 24 h. After 72 h, cytotoxicity was measured using a modified version of the VITAL-FR assay to asses killing of MHC-deficient K562 target cells versus killing of HLA-A3-transduced K562 cells as negative control target cells. This assay has been detailed described elsewhere^33^ and more information is provided in the Supplementary Material.

### MDSC and Treg staining assay

These cell subsets were measured by flow cytometry. Staining details are provided in the Supplementary Material.

### Statistics

All statistics were done with paired student's *t*-test assuming normal distribution.

## Results

To elucidate the immunological effects of 5-Azacytidine treatment, we collected blood samples from MDS, AML and CMML patients before and after each 5-Azacytidine treatment cycle for at least three cycles.

### 5-Azacytidine enhances T-cell mediated tumor cell recognition

**Figure 1:**
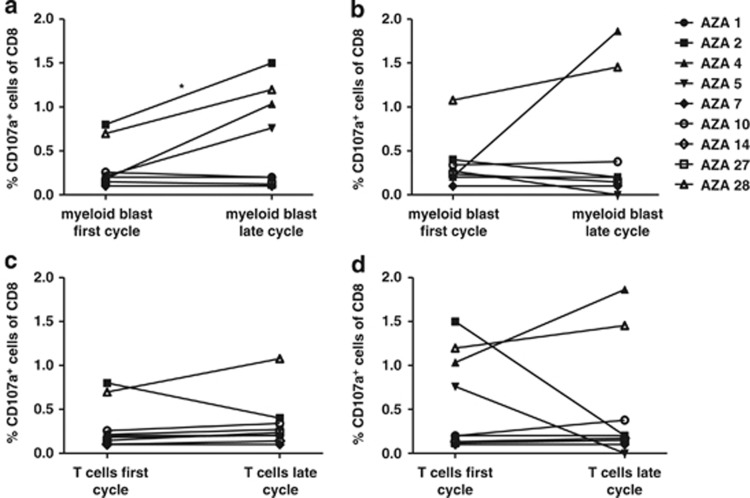
Enhanced direct *ex vivo* cytotoxicity in patients treated with 5-Azacytidine. CD8 T-cell reactivity upon co-culture with CD34 myeloid blasts is depicted, measured by CD107a expression on the CD8 T cells. T cells and myeloid blasts were isolated from a first cycle sample (pre-treatment) and from a late cycle (4th-6th cycle) sample, separated and co-cultured in four combinations. (**a**) First cycle T cells against first and late cycle myeloid blasts. (**b**) Late cycle T cells against first and late cycle myeloid blasts. (**c**) First and late cycle T cells against first cycle myeloid blasts. (**d**) First and late cycle T cells against late cycle myeloid blast cells. Note that AZA 14 is only included in (**c**, **d**). The frequency of CD107a T cells are given in percentage of CD8 T cells. Significance is indicated by *P<0.05.

We investigated the direct CD8 T-cell mediated effect of 5-Azacytidine treatment on tumor cell recognition throughout therapy. CD34 was used as a surrogate marker for myeloid blasts. We separated CD8 T cells and CD34 myeloid blasts, rested them overnight and re-exposed T cells to tumor cells from different time points to measure cytotoxic recognition by CD8 T cells. By this design, it was possible to separately examine the effect of 5-Azacytidine on the T-cell subset and on the tumor cell subset. This was done by keeping one of the variables constant, in terms of drug exposure of the cells or not. Cytotoxic activity was measured based on surface expression of the degranulation marker CD107a on CD8 T cells (gating strategy shown in Supplementary Figure S1).^29^ We observed an induction in CD107a-positive CD8 T cells when pre-treatment T cells were exposed to treatment-affected tumor cells as opposed to pre-treatment tumor cells (*P*<0.05) (Figure 1a). However, no statistically differences were observed when T cells from a late cycle were exposed to either pre- or post-treatment tumor cells (Figure 1b), although in two patients (AZA 4 and AZA 28) there was an increase in tumor-cell recognition also by these post-treatment T cells. If instead focusing on possible 5-Azacytidine-mediated induction of tumor-specific CD8 T cells, we observed no difference in T-cell reactivity between the pre- and post-treatment time points, neither when exposed to pre- nor post-treatment tumor cells (Figures 1c and d, respectively). Thus, as measured in this assay, tumor cells were more efficiently recognized by T cells when treated with 5-Azacytidine *in vivo,* while the T-cell compartment was not significantly affected by the treatment.

### 5-Azacytidine induces transient increase in cancer-testis antigen-specific T cells

**Figure 2:**
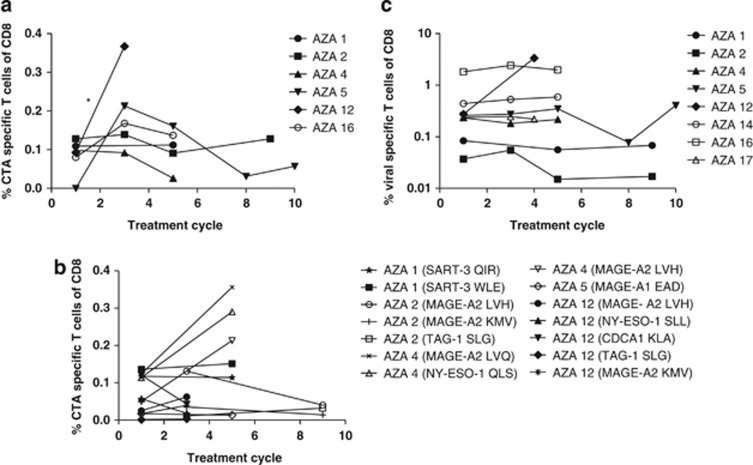
Cancer testis antigen (CTA)-specific T cells in the peripheral blood of patients. Detection of CTA- or viral-specific T cells in PBMCs by MHC multimers, expressed in percentage of CD8 T cells. (**a**) The sum of CTA-specific T cells as measured in peripheral blood at different time points during treatment. Eight patients were tested, results from the six patients with detectable responses are shown. First cycle represents a sample obtained before treatment. The following responses were found for each patient: AZA 1 (SART-3_WLE_, SART-3_QIR_, Sp17_ILD_), AZA 2 (MAGE-A2_LVH_, MAGE-A2_KMV_, TAG-1_SLG_), AZA 4 (MAGE-A2_LVH_, MAGE-A2_LVQ_, NY-ESO-1_QLS_), AZA 5 (MAGE-A1_EAD_), AZA 12 (MAGE-A2_LVH_, MAGE-A2_KMV_, CDCA1_KLA_, TAG-1_SLG_, NY-ESO-1_SLL_, MAGE-A1_EAD_) and AZA 16 (MAGE-A2_LVH_, MAGE-A2_KMV_, GnTV_VLP_, TAG-1_SLG_). (**b**) The frequency of individual CTA-specific T cells detected after an *in vitro* peptide pre-stimulation was performed at different time points during treatment. (**c**) The sum of virus-specific T cells detected over the course of treatment. The following responses were detected: AZA 1 (EBV_RLR_, EBV_RLR_, FLU_ILR_), AZA 2 (EBV_GLC_, FLU_ILR_), AZA 4 (EBV_GLC_, EBV_YVL_, FLU_GIL_), AZA 5 (FLU_BP-VSD_), AZA 12 (CMV_VTE_, CMV_YSE_, CMV_NLV_, FLU_GIL_), AZA 14 (CMV_YSE_, CMV_VTE_, FLU_BP-VSD_), AZA 16 (CMV_NLV_, EBV_GLC_) and AZA 17 (CMV_YSE_, CMV_VTE_, FLU_BP-VSD_). MHC-multimer-specific T cells are given in percentage of CD8 cells. Significance is indicated by *P<0.05.

We further investigated whether CTA-specific T-cell responses could be detected in peripheral blood from patients and whether the frequency of these were induced during 5-Azacytidine treatment. We measured CTA-specific T cells using a panel of MHC multimers with 43 CTA-derived T-cell epitopes restricted to HLA-A1 (4 epitopes), -A2 (32 epitopes), -A3 (4 epitopes) or -B7 (3 epitopes) (details are provided in Supplementary Table S2). To enable screening of T-cell reactivity against this large panel of epitopes, we used a combinatorial encoding principle for MHC multimer-based detection of specific T-cell populations by flow cytometry. This method enabled us to analyze for up to 27 different specificities in each sample (two examples of this staining method are provided in Supplementary Figure S2).^19, 30^ CTA-specific T-cell responses were found in six of eight patients analyzed. We detected responses against 13 different T-cell epitopes derived from 9 different CTAs: SART-3, MAGE-A1, MAGE-A2, TAG-1, NY-ESO-1, NUF3, GnTV, CDCA1 and Sp17 (individual frequencies are shown in Table 2, compiled frequencies of CTA-specific T cells per patient are shown in Figure 2a). Responses were detected in low frequencies both before and during treatment. We measured a transient increase in CTA-specific T cells (*P*<0.05, first cycle versus first measured treatment time point), followed by stabilization or declining levels in peripheral blood at later cycles. To further boost these low-frequent responses and confirm the early rise in T-cell reactivity against CTA-derived epitopes, we performed an *in vitro* peptide-stimulation including 14 of the 16 responses detected directly *ex vivo* in patients AZA 1, 2, 4, 5 and 12. T-cell responses found in AZA 16 were not tested due to lack of material. Moreover, from these *in vitro* stimulated cultures we observed a tendency of increase in specific T cells from pre-treatment to the first measured treatment time point (at 3rd or 5th treatment cycle) (Figure 2b, representative dot plot examples are shown in Supplementary Figure S3). In addition, we measured the frequency of virus-specific CD8 T cells to assess whether the transient increase in CTA-specific T cells was mediated by a general immune modulatory effect, but no changes in virus-specific T-cell frequencies were detected over the course of treatment (Figure 2c). Thus, CTA-specific T cells are present in MDS, AML and CMML patients, and, although the frequency is low, there is a significant increase specifically affecting these T-cell populations during 5-Azacytidine treatment.

### 5-Azacytidine only modestly affects general populations of immune effector cells

**Figure 3:**
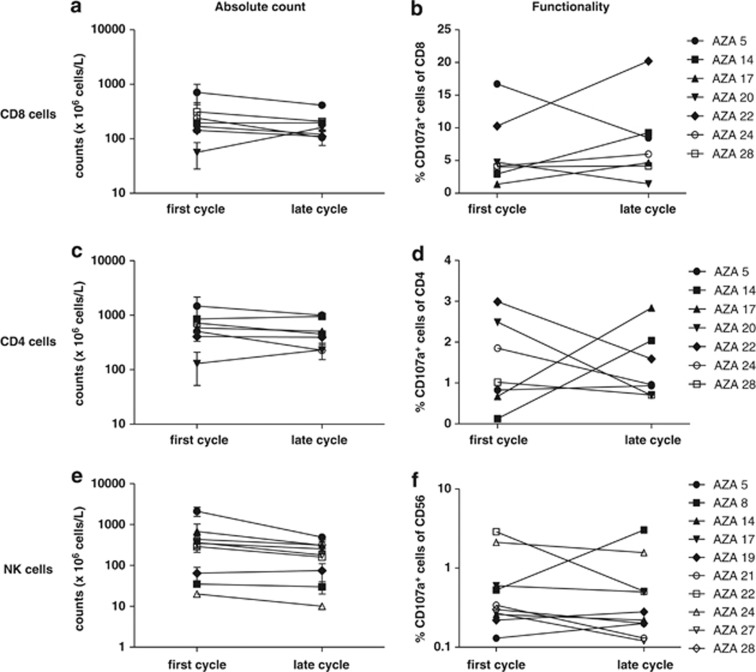
General immune effector cells are not affected by 5-Azacytidine treatment *in vivo*. The number and reactivity of NK cells and CD8 and CD4 T cells are shown. First cycle represents a sample obtained before treatment. (**a**, **c**, **e**) absolute peripheral blood counts of CD8 and CD4 T cells and CD3^−^CD56^+^CD16^+/−^ NK cells, respectively. (**b**, **d**, **f**) Expression of CD107a on CD8 and CD4 T cells and NK cells, respectively, in response to SEB (for T cells) or to K562 cells (for NK cells).

The increased immunogenicity of tumor cells observed after 5-Azacytidine treatment and the increased frequency of CTA-reactive T cells points to an ideal combination of 5-Azacytidine with immunotherapy to further boost the T-cell arm. However, before initiating such measures, any potential immune modulatory effect of 5-Azacytidine on both stimulatory and inhibitory immune cell subsets needs to be elucidated. We measured *ex vivo* total counts and reactivity of CD4 and CD8 T cells and NK cells. We used polyclonal activation of CD4 and CD8 T cells through the super-antigen *Staphylococcus aureus* enterotoxin B (SEB) and activated NK cells upon mix with K562 cells. As a signature of release of cytolytic granules, we measured surface expression of CD107a. For each patient, we investigated four time points: first and last day in the first cycle and first and last day in a late cycle. Blood from first cycle, first day was obtained before treatment. We found no significant differences in either subset within cycles (data not shown). Therefore, in subsequent analyses we pooled measurements from first and last day within the same cycle as a pseudo duplicate to investigate the effect of long-term treatment with 5-Azacytidine. No significant differences in the CD4 or CD8 T cell or the CD56^+^CD16^+/−^ NK-cell populations were found between first and late cycle in neither absolute counts nor in the frequency of CD107a-expressing cells upon SEB or K562 stimulation, respectively (Figures 3a–f).

### 5-Azacytidine affects NK cells *in vitro* and *in vivo*

**Figure 4:**
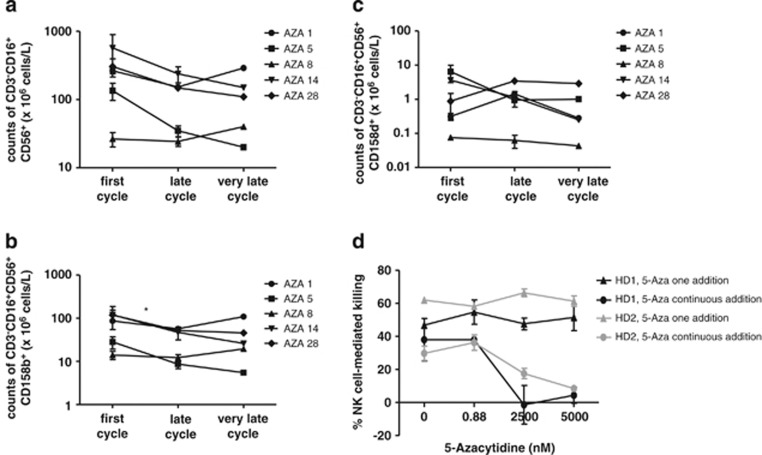
Functional capabilities of NK cells affected by 5-Azacytidine *in vivo* and *in vitro*. The number and functional activity of NK cells, when further divided in subsets and measured over a longer treatment period, and the *in vivo* and *in vitro* impact of 5-Azacytidine on NK-cell functionality. (**a**) Absolute peripheral blood counts of CD3^−^CD56^+^CD16^+^ NK cells, *P*=0.061 for first versus late cycle. (**b**) Absolute peripheral blood counts of NK cells with the inhibitory phenotype CD3^−^CD56^+^CD16^+^CD158b^+^. (**c**) Absolute peripheral blood counts of NK cells with the activating phenotype CD3^−^CD56^+^CD16^+^CD158d^+^. (**d**) NK cell-mediated killing of K562 cells after 5-Azacytidine addition, either once, or every 24 h (circles, 5-Aza continuous addition) or only at the initiation of the 72 h culturing period (triangles, 5-Aza one addition). Analyses were performed on two healthy donors (black and gray symbols, respectively). K562 killing was determined by a flow cytometry-based NK cell-killing capacity assay. NK cell-mediated killing of K562 cells was compared to a negative control with no effector cells present and killing of a HLA-A3 transduced K562 line. Counts are given in 10^6^ cells/l of blood. First cycle represents samples obtained before treatment. Significance is indicated by *P<0.05.

Previous *in vitro* studies suggested that 5-Azacytidine treatment may hamper NK-cell reactivity,^23, 24^ and, to further elucidate any changes in the NK-cell subset, we extended the measurement of NK-cell counts and functionality to a very late sample (10th cycle). Moreover, we directed the measurement specifically to the CD56^+^CD16^+^ NK cells and measured the surface-expression of two functional NK receptors, the inhibiting CD158b/NKAT2 and the activating CD158d/KIR2DL4 receptor, inspired by these same *in vitro* studies.^23, 24^ These functional receptors were included to further describe the effect of *in vivo* treatment with 5-azacytidine. As no significant decrease in CD56^+^ NK cell numbers was found (Figures 3e–f), we speculated that the phenotype of these could be altered and thus enhancing or diminishing the anti-tumor efficacy. In these experiments, we observed a tendency to decrease in absolute counts of the general CD56^+^CD16^+^ NK-cell population (*P*=0.061) and a significant decline specifically of the NK-cell subset expressing the inhibitory receptor CD158b (*P*<0.05, Figures 4a and b). In contrast, no changes were observed in the NK-cell subpopulation expressing the activating receptor CD158d (Figure 4c). It should be noted, however, that the frequency of CD56^+^CD16^+^CD158b^+^ NK cells in the total CD56^+^CD16^+^ NK-cell population remained stable. No changes were found from the late (4th to 6th cycle) to the very late (10th cycle) samples. To directly test whether 5-Azacytidine modulates the killing capacity of NK cells *in vitro*, we isolated NK cells from two healthy donors and rested them overnight. We then added 5-Azacytidine in three different concentrations and cultured the cells for 72 h either with consecutive addition of 5-Azacytidine every 24 h or not. The three concentrations used were 0.88, 2500 nM or 5000 nM, representing the 8-hour physiological concentration (calculated from Marcucci *et al.*^34^), and the two concentrations used in previous *in vitro* studies, respectively.^23, 24^ No impairment of the NK cell-mediated killing even at the highest concentration could be observed when 5-Azacytidine was added only once (Figure 4d). Similarly, no killing impairment was observed after re-addition of 5-Azacytidine every 24 h at 0.88 nM, but a significant decrease in killing capacity was found for cells affected with 2500 nM or 5000 nM 5-Azacytidine, *P*=0.001 and *P*=0.04, respectively. Thus, there is a clearly concentration-dependent inhibition of NK cell-mediated killing capacity by 5-Azacytidine.

### 5-Azacytidine does not affect inhibitory immune cells

**Figure 5:**
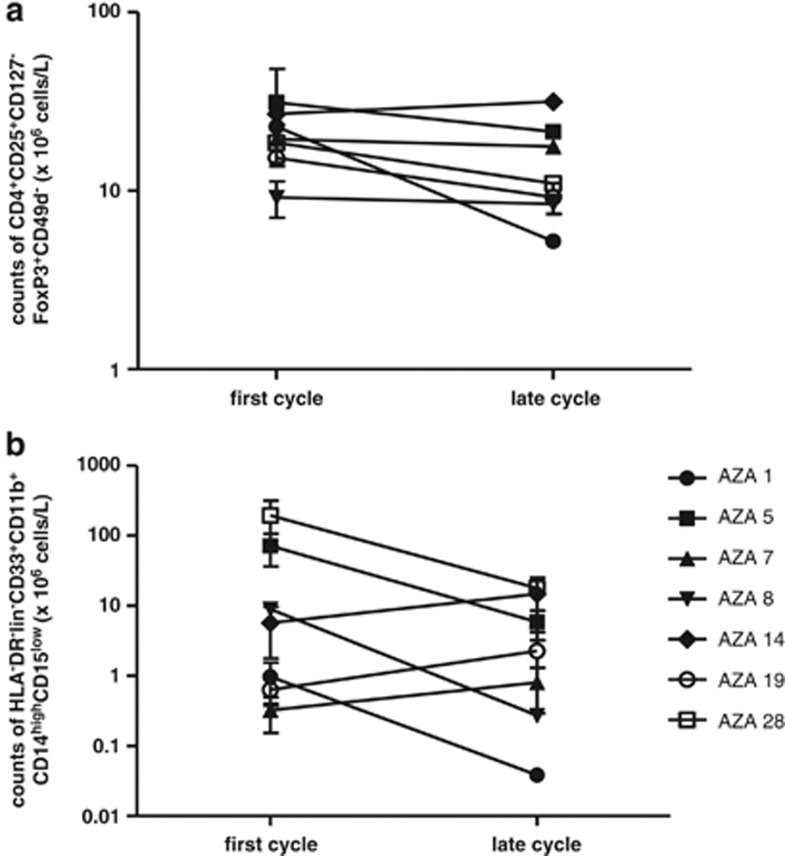
Inhibitory cell subsets are not affected by 5-Azacytidine treatment *in vivo*. Analyses of Tregs and monocytic MDSCs during 5-Azacytidine treatment are shown. (**a**) Absolute peripheral blood counts of CD4^+^CD25^+^CD127^−^FOXP3^+^CD49d^−^ Tregs over the course of treatment. (**b**) Absolute peripheral blood counts of CD3^−^CD19^−^CD56^−^HLA-DR^−^CD33^+^CD11b^+^CD14^high^CD15^low^ monocytic MDSCs over the course of treatment. Counts are given in 10^6^ cells/l of blood. First cycle represents samples obtained before treatment.

To investigate whether 5-Azacytidine influences the immune suppressive cell types, and thereby possible exert a general immune inhibitory effect, we monitored the absolute count of Tregs and monocytic MDSCs, phenotypically defined as CD4^+^CD25^+^CD127^−^FOXP3^+^CD49d^−^ and CD3^−^CD19^−^CD56^−^HLA-DR^−^CD33^+^CD11b^+^CD14^high^CD15^low^, respectively, before and after 5-Azacytidine treatment (Figure 5). Although patients had variable levels of monocytic MDSCs and Tregs, cell counts for both cell populations remained stable upon 5-Azacytidine treatment.

## Discussion

5-Azacytidine treatment has proven survival benefit in selected groups of MDS and AML patients, but the mechanism of action is only partly understood. It has been shown that 5-Azacytidine upregulates tumor suppressor genes by demethylation^35, 36^ and also CTAs that are known targets for immunological recognition of cancer cells are upregulated.^8, 9, 10^ We show here that CD34 myeloid cells (as a surrogate marker for malignant blasts) were significantly better recognized by host T cells after 5-Azacytidine treatment. This seemed to be an effect of increased tumor cell visibility rather than induced T-cell reactivity. The same mechanism likely occurs directly at the tumor site and thus may contribute to the clinical efficacy of the drug *in vivo*. On the basis of our analyses, the enhanced CD8 T-cell recognition is the predominant immune-related mechanism, as only minor effects on the NK cells and no significant changes in the general CD4 and CD8 T-cell populations were observed. Numerous mechanisms could be responsible for this increased tumor recognition. The upregulation of CTAs is a likely explanation, but is not formally correlated to the induced immune recognition. In addition, modulation of inhibitory receptors on tumor cells interacting with host T cells could be affected. A previous study showed the presence of programmed cell death 1 ligand (PD-L1) on the surface of leukemic cells *ex vivo*, and thus this marker may have a role in regulating the immune response.^37^

It was previously reported that 5-Azacytidine-induced upregulation of CTAs correlated with better T-cell recognition of tumor cells *in vitro.*^8^ In addition, *in vivo,* CTA-specific T cells have been found after treatment with 5-Azacytidine, but not correlated with recognition of tumor.^9, 10^ As has been demonstrated here, along with enhanced recognition of tumor cells, we observed an increase in T cells recognizing a large panel of CTAs after initiation of 5-Azacytidine treatment.

Along with enhanced tumor recognition, we analyzed for induction of CTA-specific T-cell responses. Because of limitations in patient material, only four patients were analyzed in both assays. Also, the enhanced tumor recognition could not be documented to increase tumor reactivity in the T-cell compartment, but rather enhanced visibility of tumor cells to pre-existing tumor-specific T cells. Thus, it is not possible to correlate the induction of CTA-specific T cells with induced tumor cell recognition in the present study. Furthermore, later during therapy the CTA-specific responses seemed to stabilize or even decline. The latter notion could possible relate to the compartment analyzed as tumor-specific T cells may traffic to the bone marrow.^38^ We have, however, no phenotypical markers included that could possible clarify this possibility. Induction of CTA-specific T cells may be more evident when 5-Azacytidine is combined with other targeted agents, such as in previous studies with the histone deacetylase inhibitor sodium valproate^10^ or the immune modulatory agent lenalidomide.^39^

We observed no general effect of 5-Azacytidine on the immune stimulatory cell subsets, CD4 and CD8 T cells and NK cells. Thus, the enhanced tumor-cell recognition and induced numbers of CTA-specific CD8 T cells together with the lack of impact on the general T-cell populations suggest that 5-Azacytidine primarily affects the tumor cells. It should be noted, however, that the 5-Azacytidine deoxyribonucleoside analog decitabine has been shown to exert a general upregulation of HLA-A1 and -A2 molecules on melanoma cell lines in addition to the upregulation of CTA expression.^40^ This could also account for the better T-cell recognition of tumor if the effect holds true for 5-Azacytidine treatment of MDS *in vivo*.

NK cells have previously been investigated in detail in relation to 5-Azacytidine and to MDS. In the present study, we found a decreasing tendency in a subpopulation of NK cells expressing the inhibitory receptor CD158b. In contrast, when NK cells were subjected to 5-Azacytidine treatment *in vitro*, we observed a remarkable decrease in killing capacity of NK cells. This effect was concentration dependent with strong inhibition induced by 2500 nM or 5000 nM, but not by 0.88 nM 5-Azacytidine when administered every 24 h. Also, no inhibition occurred when 5-Azacytidine was given as a single exposure in all three doses. Clinically, patients were treated for 5 days every fourth week, leading to peak plasma concentrations of around 3 μM, and as 5-Azacytidine is promptly degraded in blood (*t*_½_, _I.V._=41 min),^34^ it will be undetectable between cycles. The systemic clearance of the drug exceeds the glomerular filtration rate,^34^ and thus the drug is expected to have a similar half life *in vitro* as *in vivo*. We did, however, not observe any functional impact on NK cells *in vivo*, although the effect is clearly evident *in vitro* in the concentration mimicking the peak plasma concentration *in vivo.* NK cells may be less vulnerable *in vivo*, or the plasma concentration is not sufficiently high during extended periods of time to confer this effect *in vivo*.

It has been reported that a number of chemotherapeutic regiments modulate the antitumor responses and especially the immune inhibitory balance in the interaction between the existent tumor-reactive T cells and the tumor cells.^20^ We analyzed the effect of 5-Azacytidine on two major inhibitory cell populations, monocytic MDSCs and Tregs, with described relevance for cancer therapy.^26^ There is still much debate on which phenotypic markers do define the human MDSCs,^41^ here we defined monocytic MDSCs as CD3^−^CD19^−^CD56^−^HLA-DR^−^CD33^+^CD11b^+^CD14^high^CD15^low^, inspired by recent published data.^41, 42^ An additional important immune inhibitory cell type and a subpopulation of the MDSCs, the polymorphonuclear MDSCs (phenotypically CD3^−^CD19^−^CD56^−^HLA-DR^−^CD33^+^CD11b^+^ CD14^low^CD15^high^), were not included in this measurement as several reports have questioned the relevance of analyzing this cell type on cryopreserved material.^43, 44^

For Tregs CD49d has been demonstrated as a valuable marker to distinguish inhibitory FOXP3 Tregs (CD49d^−^) from the pro-inflammatatory subset (CD49d^+^),^45^ thus we defined Tregs as CD4^+^CD25^+^CD127^−^FOXP3^+^CD49d^−^. No effects were observed on either Tregs or monocytic MDSCs in peripheral blood over the course of treatment. However, a possible local effect around clusters of myeloid blasts in bone marrow cannot be excluded, as no bone marrow samples were available for analyses in the present study. Such a local effect has been observed for both solid tumors and AML in relation to other chemotherapy regiments.^20^ 5-Azacytidine's effect on Tregs may differ dependent on the treatment context, as it has been reported that 5-Azacytidine increases the number of Tregs in a patient group receiving allogeneic stem cell transplantation.^46^ However, another recent study showed decreased Treg numbers upon treatment with 5-Azacytidine, although this was only investigated in one patient.^47^ Combination of immune therapy and chemotherapy have by several lines of data been suggested to be synergistic.^20, 42^ A recent report showed that induction of antitumor immunity is especially effective when combining chemotherapy with a high antigen load in the tumor^48^—this may speak for the successful application of 5-Azacytidine in combination with immune therapy, because of the evident induction of tumor-associated antigen expression. Furthermore, several strategies for therapeutic vaccination in patients with AML and MDS have been tested in phase 1/2 studies, indicating that vaccination may be beneficial in this patient group.^49, 50, 51, 52^ Combination therapy with 5-Azacytidine and vaccination may potentially provide synergistic effects with clinical benefit for patients with MDS, AML and CMML.

## Figures and Tables

**Table 1:** Overview of 17 included patients with high-risk MDS or MDS with high risk features

*AZA no.*	*Age at diagnosis*	*Sex*	*Diagnosis*	*Cycles received*
AZA 1	79	f	MDS	14
AZA 2	63	m	MDS	9
AZA 4	73	m	AML	6
AZA 5	78	f	CMML	41
AZA 7	39	m	MDS	9
AZA 8	64	m	MDS	12
AZA 10	84	m	CMML	16
AZA 12	78	m	AML	3
AZA 14	77	f	MDS	12
AZA 16	76	m	MDS	4
AZA 17	70	f	AML	3
AZA 19	67	f	AML	10
AZA 20	75	f	MDS	9
AZA 22	62	m	MDS	10
AZA 24	73	f	MDS	4
AZA 27	76	m	MDS	26
AZA 28	74	m	MDS/AML	12

Abbreviations: AML, acute myeloid leukemia; CMML, chronic myelomonocytic leukemia; f, female; m, male; MDS, myelodysplastic syndrome.

**Table 2:** CTA-specific T-cell responses during AZA treatment, direct *ex vivo*, in % of CD8 cells

*Patient no.*	*Specificity*	*Cycle no.1*	*Cycle no.3*	*Cycle no.5*	*Cycle no. 8*	*Cycle no.9*	*Cycle no. 10*
AZA 1	SART- 3 WLE	0.019	ND	0.025	ND	ND	ND
	SART- 3 QIR	0.052		0.037			
	Sp17 ILD	0.038		0.050			
AZA 2	MAGE-A2 LVH	0.072	0.065	0.041	ND	0.050	ND
	MAGE-A2 KMV	0.020	0.018	0.012		0.019	
	TAG-1 SLG	0.036	0.056	0.038		0.059	
AZA 4	MAGE-A2 LVH	0.080	0.086	0.020	ND	ND	ND
	MAGE-A2 LVQ	0.060	0.002	0.001			
	NY-ESO-1 QLS	0.012	0.004	0.005			
AZA 5	MAGE-A1 EAD	0.001	0.213	0.161	0.031	ND	0.057
AZA 12	MAGE-A2 LVH	0.031	0.030	ND	ND	ND	ND
	MAGE-A2 KMV	0.026	0.015				
	CDCA1 KLA	0.007	0.003				
	TAG-1 SLG	0.019	0.044				
	NY-ESO-1 SLL	0.007	0.010				
	MAGE-A1 EAD	0.003	0.265				
AZA 16	MAGE-A2 LVH	0.042	0.100	0.059	ND	ND	ND
	MAGE-A2 KMV	0.013	0.025	0.009			
	TAG-1 SLG	0.023	0.042	0.068			
	GnTV VLP	0.002	0.001	0.001			

Abbreviation: ND, not determined.

## Supplementary Material

Supplementary Figure S2

Supplementary Figure S2 revised
