# Supplementary figures and images for: 5-Azacytidine treatment sensitizes tumor cells to T-cell mediated cytotoxicity and modulates NK cells in patients with myeloid malignancies
Source: Blood Cancer J. 2014 Mar 28;4(3):e197–. doi: 10.1038/bcj.2014.14 (PMC3972700; doi:10.1038/bcj.2014.14)

## Slide 1
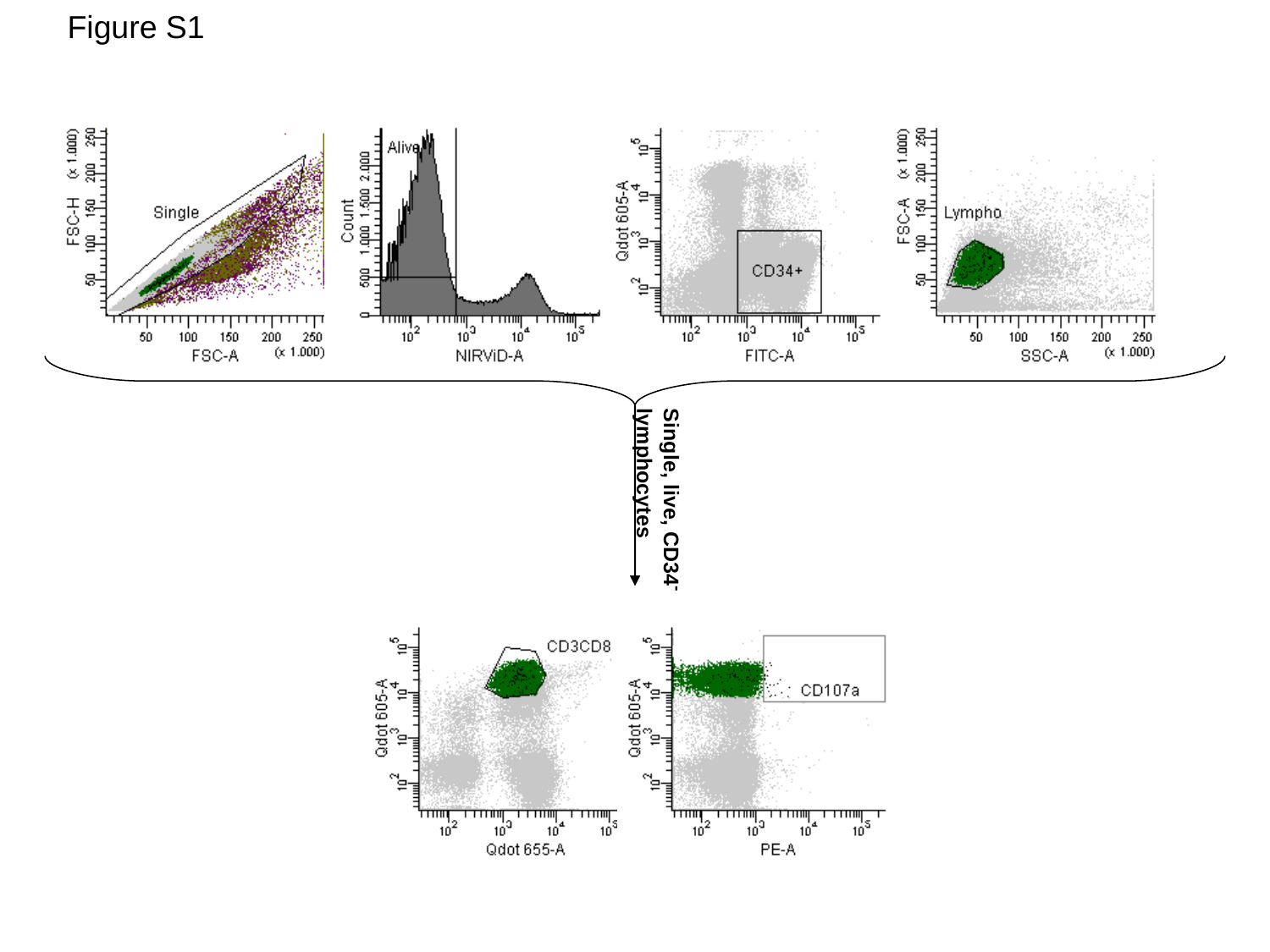

Figure S1
Single, live, CD34- lymphocytes

Supplement: Supplementary Figure S1 [file bcj201414x1.ppt]

## Slide 1
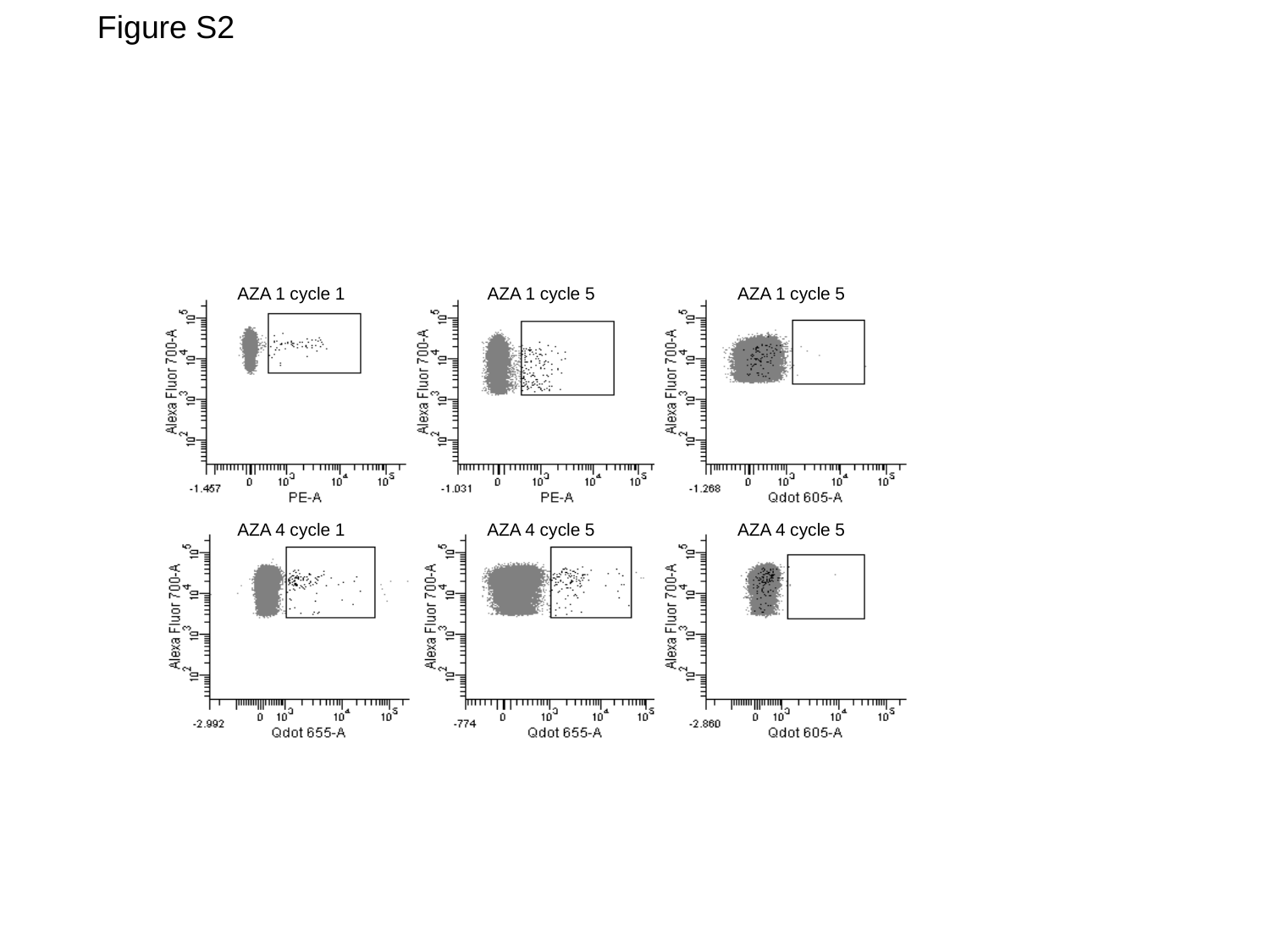

Figure S2
AZA 1 cycle 1
AZA 1 cycle 5
AZA 1 cycle 5
AZA 4 cycle 5
AZA 4 cycle 5
AZA 4 cycle 1

Supplement: Supplementary Figure S2 [file bcj201414x2.ppt]

## Slide 1
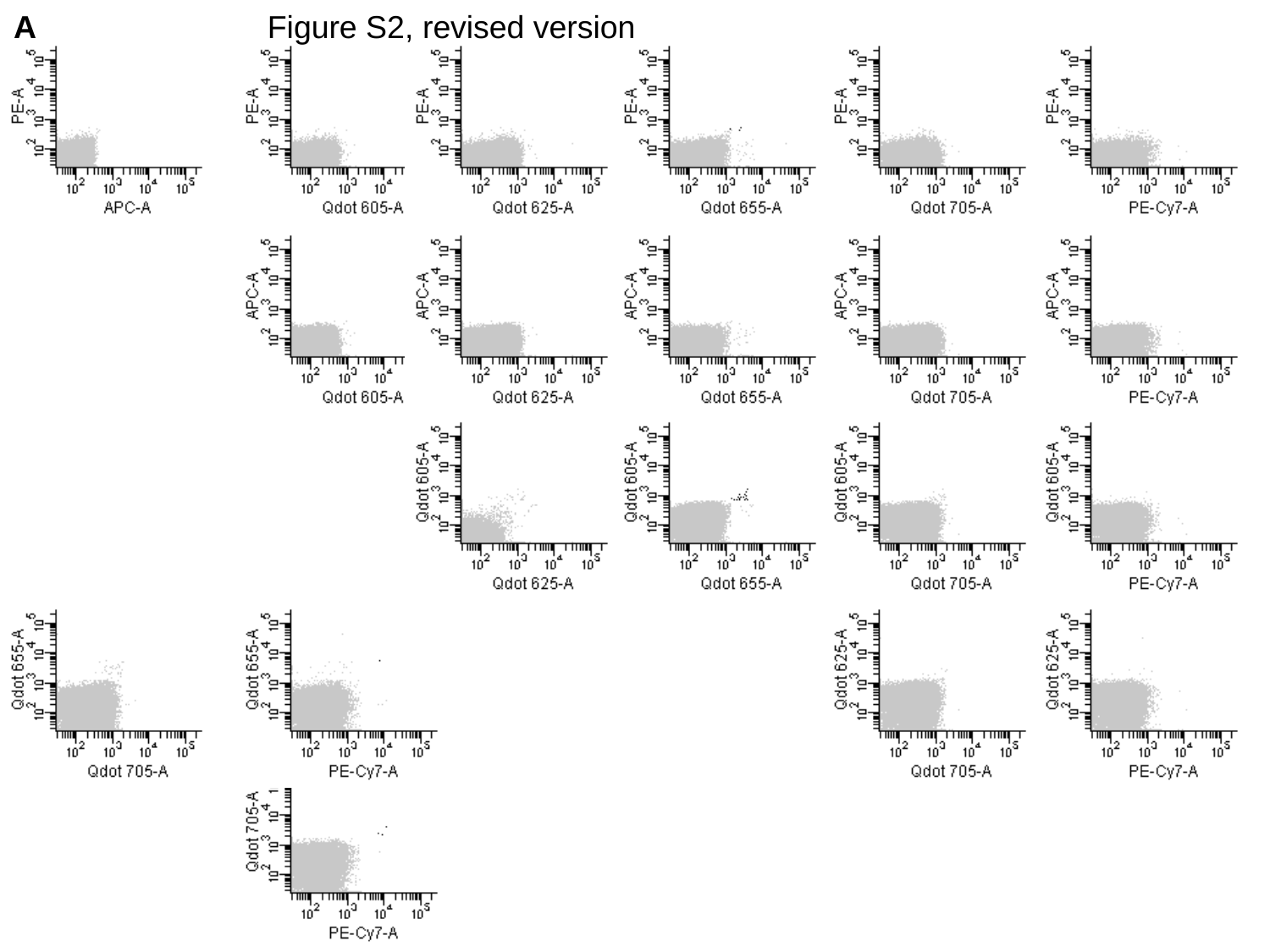

A
Figure S2, revised version

## Slide 2
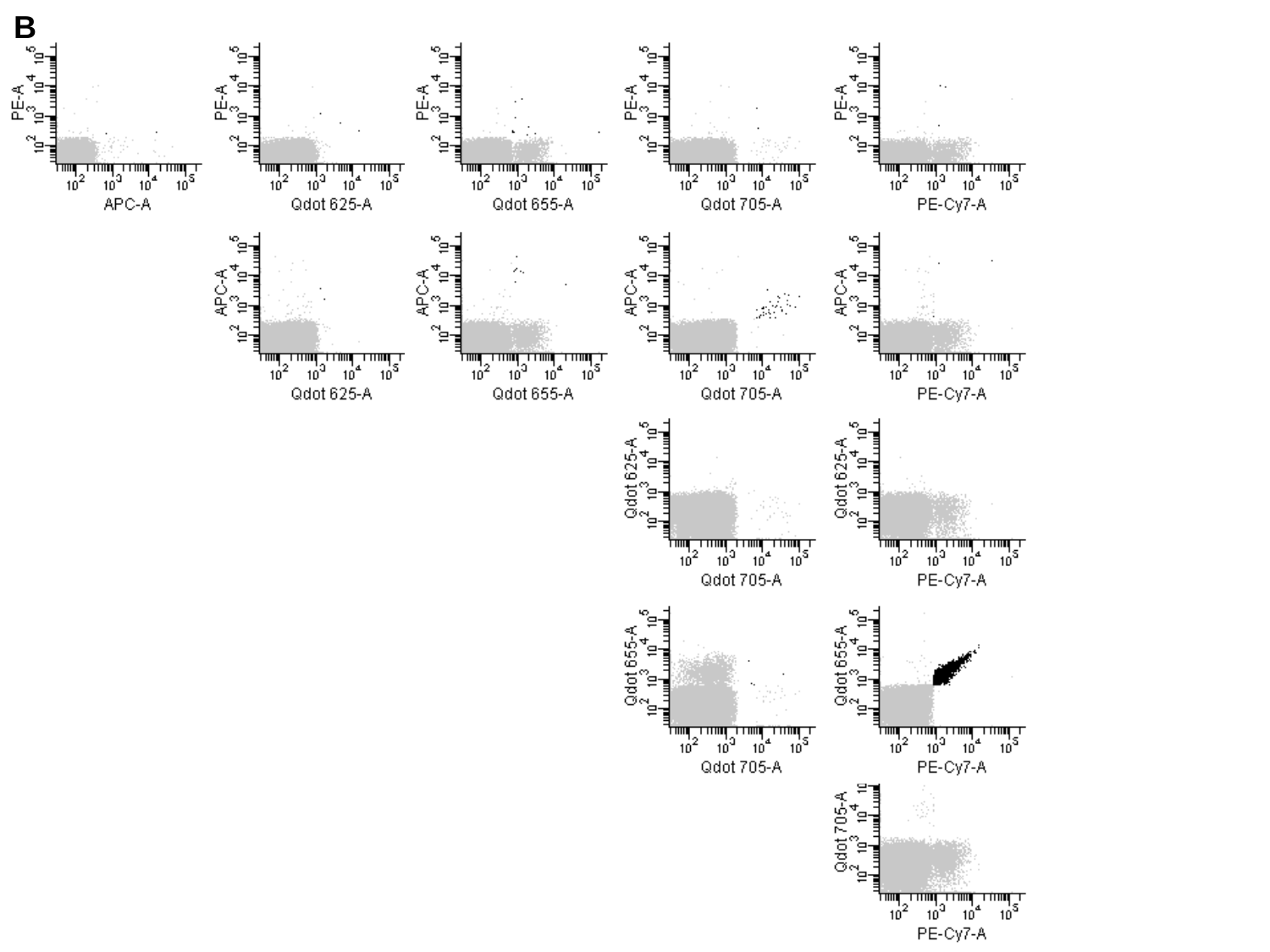

B

Supplement: Supplementary Figure S2 revised [file bcj201414x3.ppt]
