# Supplementary material for: 5-Azacytidine treatment sensitizes tumor cells to T-cell mediated cytotoxicity and modulates NK cells in patients with myeloid malignancies
Source: Blood Cancer J. 2014 Mar 28;4(3):e197–. doi: 10.1038/bcj.2014.14 (PMC3972700; doi:10.1038/bcj.2014.14)
Supplement: Supplementary Figure legend [file bcj201414x4.doc]

**Figure legends, supplemental figures**

**Figure S1.** Gating strategy for the staining of CD8 and CD34 mix is shown.

These data were obtained after staining of the mix of CD34 myeloid blasts obtained prior to treatment (1-1) and CD8 T cells obtained at a late cycle (5-1) from patient AZA 28 (data also shown in figure 1 B-C). From the CD34 plot an inverse gate was formed and combined with gates in the three remaining plots to form the “single, live, CD34 negative lymphocytes”. CD3CD8 cells are gated in the plot bottom left and the remaining plot shows the gating on CD107a cells within the CD3CD8 population.

**Figure S2.** Dot plots examples from two experiments with combinatorial encoding of MHC multimers for antigen specific T-cell detection in PBMCs. Only events negative for all multimers (gray) or positive for exactly two multimers (black) are shown. A) AZA 2, cells obtained after cycle 5. 0.02% of the CD8 cells are specific for the HLA-A2 MAGE-A2_KVM_ epitope observed in the Qdot605/Qdot655 combination. The black dot in the PE/Qdot655 and Qdot705/PE-Cy7 combinations are below threshold for detection. B) AZA 16, cells obtained prior to treatment. 0.041% of the CD8 cells are specific for the HLA-A2 MAGE-A2_LVH_ epitope seen in the APC/Qdot705 combination and a T-cell response on 2.4% of the CD8 cells specific for a virus-derived epitope is seen in the Qdot655/PE-Cy7 combination. The remaining black dots are below threshold for detection. Further information about the method of combinatorial encoding of MHC multimers is found in references 19 and 30.

**Figure S3.** Examples of flow cytometry dot plots identifying CTA-specific T-cell populations. Responses are shown from two time-points (first cycle and cycle 5) for two patients with CTA-specific T-cell responses (AZA 1: SART-3_QIR_ and AZA 4: NY-ESO-1_QLS_) after *in vitro* peptide pre-stimulation. Plots to the right represent negative controls using a HLA-A2-HIV_ILK_ MHC multimer.
