# Supplementary material for: 5-Azacytidine treatment sensitizes tumor cells to T-cell mediated cytotoxicity and modulates NK cells in patients with myeloid malignancies
Source: Blood Cancer J. 2014 Mar 28;4(3):e197–. doi: 10.1038/bcj.2014.14 (PMC3972700; doi:10.1038/bcj.2014.14)
Supplement: Supplementary Figure S2 [file bcj201414x5.doc]

Figure S2**:**

Dot plots from two combinatorial encoding experiments with PBMCs, only cells negative for all multimers or more than three multimers (gray) or positive for exactly two multimers (black) are shown. A) AZA 2, cells obtained after cycle 5. 0.0221% of the CD8 cells are specific for the HLA-A2 MAGE-A2 KVM epitope seen in the Qdot605/Qdot655 combination. The black cells seen in the PE/Qdot655 and Qdot705/PE-Cy7 combinations are considered background. B) AZA 16, cells obtained prior to treatment. 0.0408% of the CD8 cells are specific for the HLA-A2 MAGe-A2 LVH epitope seen in the APC/Qdot705 combination and a T-cell response on 2.4270% of the CD 8 cells specific for a virus epitope is seen in the Qdot655/PE-Cy7 combination. The remaining black cells in different plots are considered background. Further information about this method of combinatorial encoding of MHC multimers is found in references 19 and 30.
