# Supplementary material for: 5-Azacytidine treatment sensitizes tumor cells to T-cell mediated cytotoxicity and modulates NK cells in patients with myeloid malignancies
Source: Blood Cancer J. 2014 Mar 28;4(3):e197–. doi: 10.1038/bcj.2014.14 (PMC3972700; doi:10.1038/bcj.2014.14)
Supplement: Supplementary Table 1 [file bcj201414x6.doc]

Supplementary Table 1: details of materials and methods according to the MIATA guideline

| **Module 1, the cell samples** | |
| --- | --- |
| **PBMC samples** | |
| Donors | MDS and AML patients and healthy volunteers from blood bank |
| Informed consent | Yes |
| Source of PBMC | Blood sample and venipuncture, leukaphereses |
| Anticoagulant | Citrat-Phosphat-Dextrose |
| Method for HLA typing | Genomic DNA typing |
| Transportation and storage | RT |
| Cell processing method | Density gradient separation |
| Dilution and washing buffer | Sterile PBS and culture medium |
| Time between blood collection and end of sample processing | < 6 hrs |
| Freezing and storage | 90% HI-FBS (Gibco, Life Technologies, Naerum, Denmark) 10% DMSO. Freezing container. Long term storage: -150°C freezer |
| N° of PBMC frozen per vial | 5 - 20 x10^6^ |
| **Cell counting and thawing** | |
| Viability after PBMC isolation | > 90% |
| Viability after thawing | 40 – 90% |
| Cell counting method | trypan blue |
| Medium used for thawing | RPMI 1640 + Glutamax, 10% HI FBS. Optional addition: 0.025 mg ml − 1 Pulmozyme (Roche, Hvidovre, Denmark) and 2.5 mM MgCl_2_ |
| Serum pretested | No |

| **Module 2, the assays** | |
| --- | --- |
| **CD8 T cells and CD34 tumor cells mix** | |
| Culture medium | X-Vivo 15 (Lonza), 10% HI-Human Serum (Sigma) |
| Isolation | MACS CD8 positive selection and CD34 negative selection kits (Miltenyi Biotec) |
| Buffer | PBS with 2% HI-FBS (Gibco) |
| **Peptide stimulation and combinatorial encoding MHC-multimer staining** | |
| Culture medium | X-vivo 15 (Lonza) with 5% HI-Human Serum (Sigma), IL-2 (20 U/ml, Proleukin Novartis) and IL-7 (5 ng/ml, Peprotech) |
| Peptide concentration for stimulation | 10 μM |
| Peptides provided by | Pepscan Ltd, NL. |
| **NK cell killing capacity assay** | |
| Isolation | MACS untouched NK cell kit followed by CD56+ kit (Miltenyi Biotec) |
| Resting medium | X-vivo 15 (Lonza) with 10% HI-Human Serum (Sigma), IL-2 (200 U/ml, Proleukin Novartis) and IL-15 (40 U/ml, Peprotech) |
| **Frequency of measured cell populations** | |
| CD8 T cell and CD34 tumor cell mix (figure 1) | - - - 1. % CD107^+^aCD8^+^ cells |
| MHC-multimer assay (figure 2) | 0.001-0.20% multimer^+^CD8^+^ direct *ex vivo*, CTA-specific T cells  0.013-0.36% multimer^+^CD8^+^ after *in vitro* peptide pre-stimulation  0.015-3.36% multimer^+^CD8^+^ direct *ex vivo*, virus-specific T cells |
| Cell counts and functionality assays (figure 3) | 56-710 x 10^6^ cells/L of CD8^+^ T cells  130-1478 x 10^6^ cells/L of CD4^+^ T cells  10-2115 x 10^6^ cells/L of CD56^+^ NK cells  1.4-20% CD107a^+^CD8^+^ cells  0.1-3.0 % CD107a^+^CD4^+^ cells  0.1-3.0 % CD107a^+^CD56^+^ cells |
| NK subpopulation and killing capacity assay analyses (figure 4) | 20-573 6 x 10^6^ cells/L of CD56^+^CD16^+^ cells  5.5-119 x 10^6^ cells/L of CD56^+^CD16^+^CD158b^+^ cells  0.043-6.5 x 10^6^ cells/L of CD56^+^CD16^+^CD158d^+^ cells  25-63% killing in effector:target ratio 10:1 |
| Tregs assay (figure 5) | 5.2-31.6 x 10^6^ cells/L of CD4^+^CD25^+^CD127^-^FoxP3^+^CD49d^-^ |
| M-MDSCs assay (figure 5) | 0.038-195 x 10^6^ cells/L of HLA-DR^-^lin^-^CD33^+^CD11b^+^CD14^high^CD15^low^ |

| **Module 3, data acquisition** | |
| --- | --- |
| Flow cytometer | BD LSR II SORP |
| Software for acquisition | Diva |
| Instrument settings and performance control | CS&T beads, daily performance check, compensation with beads |
| Number of cells acquired | All in the tube |
| Lasers and filters | LSRII: UV laser (355nm, 60 mW): detector A: 710/50 and 680LP, detector B: 605/12 and 595LP, detector C: 580/30. Remaining detectors empty. Violet laser (405nm, 100 mW): detector A: 655/6 and 635 LP, detector B: 625/20 and 610 LP, detector C: 450/50. Blue laser (488 nm, 100 mW): detector A: 780/60 and 735 LP, detector B: 710/50 and 685LP, detector C: 670/30 and 635LP, detector D: 610/20 and 600LP, detector E: 585/15 and 570LP, detector F: 525/50 and 505LP, detector G: 488/10. Yeloow green laser (561 nm, 50 mW): detector A: 800/30 and 770LP, detector B: 695/40, detector C empty. Red laser (640 nm, 40 mV): detector A: 780/60 and 735 LP, detector B: 725/50 and 710 LP, detector C: 660/20.  FACSCanto II: Violet laser (405 nm, 25 mW): detector A: 510/50 and 502LP, detector B: 450/50, detector C empty. Blue Laser (488 nm, 20 mW): detector A: 780/60 and 735LP, detector B: 670LP and 655LP, detector C: 610LP, detector D: 585/42 and 556LP, detector E: 530/30 and 502LP, detector F: 488/10. Red laser (633 nm, 17mW): detector A: 780/60, detector B: 685LP, detector C: 660/20.  Filters were produced by BD Biosciences and were changed according to daily performance check. |

| **Module 4, data processing** |  |
| --- | --- |
| Software for analysis | Diva Software v6.1.3 |
| Gating strategy for measurements of viability and CD8^+^ cells | singlets FSC-A/H or FSC-A/W, lymphos FSC-A/SSC-A, living lymphos FSC-A/dead cell dye (gate). Example on gating strategy is shown in figure S1. |
| Gating between experiments | One mastergate set in comparison analyses. |
| Any data excluded | No |
| Positivity criteria | n.a. |
| Raw data provided on demand | Yes |

| **Module 5, Lab conditions** | |
| --- | --- |
| Guidance of lab operations | Exploratory research |
| Trained personal | Yes |
| Accreditation of the lab | No |
| Participation to proficiency panels | Yes, CIP |
| Status of protocols | Established lab protocols |
| Status of assays | Qualified |

FBS = fetal bovine serum; HI = heat- inactivated; n.a. = not applicable; RT = room temperature; PBS: phosphate buffer; CTA: cancer-testis antigen; LP: longpass filter; FCS: forward scatter; SSC: side scatter; CIP: CIMT (The Association for Cancer Immunotherapy) Immunoguiding program
