# Supplementary material for: 5-Azacytidine treatment sensitizes tumor cells to T-cell mediated cytotoxicity and modulates NK cells in patients with myeloid malignancies
Source: Blood Cancer J. 2014 Mar 28;4(3):e197–. doi: 10.1038/bcj.2014.14 (PMC3972700; doi:10.1038/bcj.2014.14)
Supplement: Supplementary Table 1 [file bcj201414x7.pdf]

Supplementary table 1: Cancer-testis antigens used for T-cell immunity screening in AZA treated patients

Gene	HLA allele	Peptide	References
CDCA1/NUF2			
	A2	KLATAQFKI	Harao, 2008
	A2	YMMPVNSEV	Harao, 2008
GnTV			
	A2	VLPDVFIRCV	Guilloux, 1996
	A2	VLPDVFIRC	Guilloux, 1996
HERV-K-MEL			
	A2	MLAVISCAV	Schiavetti, 2002
LAGE-1			
	A2	MLMAQEALAFL	Aarnoudse, 1999
	A2	SLLMWITQC	Rimoldi, 2000
	B7	APRGVRMAV	Slager, 2004
MAGE-A1			
	A1	EADPTGHSY	Traversari, 1992
	A2	KVLEYVIKV	Ottaviani, 2005; Pascolo, 2001
	B7	RVRFFFPSL	Luiten, 2000
	A3	SLFRAVITK	Chaux, 1999
MAGE-A10			
	A2	GLYDGMEHL	Bricard, 2005; Huang, 1999
MAGE-A2			
	A2	KMVELVHFL	Visseren, 1997
	A2	YLQLVFGIEV	Kawashima, 1998
	A2	LVHFLLLKY	Bredenbeck. 2005
	A2	LVQENYLEY	Bredenbeck. 2005
MAGE-A3			
	A1	EVDPIGHLY	Gaugler, 1994
	A2	FLWGPRALV	van der Bruggen, 1994
	A2	KVAELVHFL	Kawashima, 1998
	A2	LVFGIELMEV	Keogh, 2001
MAGE-A4			
	A1	EVDPASNTY	Kobayashi, 2003
	A2	GVYDGREHTV	Duffour, 1999
MAGE-A6			
	A2	YLEYRQVPV	Graff-Dubois, 2002

Gene	HLA allele	Peptide	References
MAGE-A9			
	A2	ALSVMGVYV	Oehlrich, 2005
MAGE-C2			
	A2	LLFGLALIEV	Ma, 2004
	A2	ALKDVEERV	Ma, 2004
NY-ESO-1 / LAG			
	A2	MLMAQEALAFL	Aarnoudse, 1999
	A2	QLSLLMWIT	Jager, 1998
	A2	SLLMWITQA	Jager, 1998; Chen, 2000
	A2	SLLMWITQC	Jager, 1998; Chen, 2000
	A2	SLLMWITQCFL	Jager, 1998
	B7	APRGPHGGAAS	Ebert, 2009
SART-3			
	A2	RLAEYQAYI	Ito, 2000
	A3	WLEYYNLER	Minami, 2007
	A3	QIRPIFSNR	Minami, 2007
	A2	LLQAEAPRL	Ito, 2000
Sp17			
	A1	ILDSSEEDK	Chiriva-Internati, 2003
SSX-2			
	A2	KASEKIFYV	Ayyoub, 2002; Bricard, 2005
	A2	RLQGISPKI	Wagner, 2003
TAG			
	A3	RLSNRLLLR	Hogen, 2004
TAG-1			
	A2	SLGWLFLLL	Adair, 2008
TRAG-3			
	A2	ILLRDAGLV	Zhu, 2003
TRP2-6b			
	A2	ATTNILEHY	Khong, 2002
