# Supplementary material for: 5-Azacytidine treatment sensitizes tumor cells to T-cell mediated cytotoxicity and modulates NK cells in patients with myeloid malignancies
Source: Blood Cancer J. 2014 Mar 28;4(3):e197–. doi: 10.1038/bcj.2014.14 (PMC3972700; doi:10.1038/bcj.2014.14)
Supplement: Supplementary Information [file bcj201414x8.doc]

**Supplementary information**

Reference numbers apply to the reference list in the main article.

General MIATA

All experiments were based on local evaluated protocols; data acquisition and data analysis were as per local resources and experience. All culture media and reagents were tested for performance before entering the protocols. In all experiments comparing proportion of cells before and after treatment (Figure 1 A-D, 3 A-F, 4 A-C, 5 A-B) “late cycle” refers to cells obtained at 4^th^-6^th^ vaccination cycle, similar to 13^th^-21^st^- week. The very late samples shown in Figure 4 A-C are from 10^th^ vaccination cycle, 37 weeks after treatment initiation. Frequency ranges of detected cell populations are provided in Table S1.

CD107a staining assay

## CD107a-PE (BD Pharmingen, Albertslund, Denmark) and BD GolgiPlug (BD Biosciences, Albertslund, Denmark) were added to the mixtures of CD34 and CD8 cells, and left incubating for 5 hours. Cells were fixed and permeabilized or not dependent on experimental setup. Cells were stained with CD3-Quantum Dot 655 (CD3-Q655, Invitrogen, Life Technologies, Naerum, Denmark), CD3-Pacific Blue or CD3-APC-Cy7 (both BD Pharmingen, Albertslund, Denmark), CD8-Q605 (Invitrogen, Life Technologies, Naerum, Denmark ) or CD8-Alexa Flour 700 (Caltag, Life Technologies, Naerum, Denmark), CD34-PerCP or CD34-FITC (both from BD Pharmingen, Albertslund, Denmark) and LIVE/DEAD® Fixable Near-IR Dead Cell Stain Kit for 633 or 635 nm excitation (Invitrogen, Life Technologies, Naerum, Denmark) or 7-Aminoactinomycin D (7-AAD, BD Pharmingen, Albertslund, Denmark). Acquisition was conducted on a FACSCanto II or LSRII (both from BD Biosciences) and data were analyzed using FACSDiva software.

MHC-multimer based flow cytometry analyses

MHC monomers for 43 CTA derived peptides restricted to HLA-A1, -A2, -A3 or -B7 (shown in Table S2) were generated using UV-induced exchange of conditional ligands, as previously described.^51,52^ MHC-multimers were generated in two-color codes for each specificity allowing parallel detection of multiple T-cell populations using a combinatorial encoding principle, as described.^19^ MHC-multimers with a HLA-A2 HIV derived peptide was used as a negative control. Peptides were purchased from Pepscan Ltd, NL.

## Approximately 1 million cells from PBMC samples or T-cell cultures were stained with MHC-multimers for 15 min at 37˚C, followed by additional incubation for 30 minutes at 4˚C with, CD3-FITC (both from BD Pharmingen, Albertslund, Denmark), and LIVE/DEAD® Fixable Near-IR Dead Cell Stain Kit for 633 or 635 nm excitation (Invitrogen, Life Technologies, Naerum, Denmark). Cells were washed and analyzed by flow cytometry. Further details can be found in a recent published protocol.^30^ Acquisition was conducted on a LSR II (BD Biosciences) and data analyzed using FACSDiva software (BD Biosciences). Gating was performed as described,^30^ the definition of positive response required 10 multimer-specific events and > 0.002% of CD8 T cells recorded.

## Vital-FR assay for measuring NK cell killing capacity

## K562 positive target cells were labeled with Cell-Trace CFSE Cell Proliferation Kit (Invitrogen, Life Technologies, Naerum, Denmark) and HLA-A3 transduced K562 negative target cells with Cell-Trace Far Red DDAO-SE (Invitrogen, Life Technologies, Naerum, Denmark). 1000 cells from each target cell population were added to each well in a 96-well plate (Corning Costar, BD Biosciences, Albertslund, Denmark) and isolated NK effector cells were mixed with target cells in the following ratios: 1000:1, 100:1, 10:1, 1:1, 0.1:1, 0.01:1 and control wells without effector cells. The assay was performed in duplicates for every 5-Azacytidine concentration for both donors, except for 1000:1 and 100:1 ratios due to lack of effector cells. Wells were re-suspended every 24 hours and tested after 72 hours; acquisition was done one a LSRII (BD Biosciences) and data were analyzed using FACSDiva Software. Data are depicted for E:T ratio 10:1.

Regulatory T-cell assay

PBMCs where thawed in RPMI 1640 + Glutamax (Sigma-Aldrich, Broendby, Denmark) with 10% FCS and DNase (Invitrogen, Life Technologies, Naerum, Denmark). The cells were surface stained with CD4-HV500 (BD Pharmingen, Albertslund, Denmark), CD127-FITC, CD25-APC (both eBioscience, AH Diagnostics, Aarhus, Denmark), CD49d-PE-Cy7 (Biolegend, Nordic Biosite, Copenhagen, Denmark) and LIVE/DEAD® Fixable Near-IR Dead Cell Stain Kit for 633 or 635 nm excitation (Invitrogen, Life Technologies, Naerum, Denmark). Intracellular staining for FOXP3-PE or iso-type control IgG-2a-PE (both eBioscience, AH Diagnostics, Aarhus, Denmark) was performed according to manufacturer’s instructions (eBiosciences). Acquisition was conducted on a FACSCanto II (BD Biosciences) and data analyzed using FACSDiva software.

MDSC assay

PBMCs where thawed in RPMI 1640 + Glutamax (Sigma-Aldrich, Broendby, Denmark) with 10% FCS (Gibco, Life Technologies, Naerum, Denmark) and DNase (Invitrogen, Life Technologies, Naerum, Denmark). Mouse serum (Trichem, Skanderborg, Denmark) was added to block Fc receptors, and cells were stained in two tubes, an IgG2a isotype control (BD Pharmingen, Albertslund, Denmark) and a surface stain for CD33-PE (Dako, Glostrup, Denmark), HLA-DR-PerCp (BD Pharmingen, Albertslund, Denmark), Lineage (CD3-PE-Cy7,CD19-Pe-Cy7,CD56-PE-Cy7), CD11b-APC (all from BD Pharmingen, Albertslund, Denmark , CD14-BV421 (Biolegend, Nordic Biosite, Copenhagen, Denmark), CD15-HV500 (BD Pharmingen, Albertslund, Denmark and NIR (Invitrogen, Life Technologies, Naerum, Denmark). Acquisition was conducted on FACSCanto II (BD) and data analyzed using FACSDiva software.
